# Real-time observations of TRIP-induced ultrahigh strain hardening in a dual-phase CrMnFeCoNi high-entropy alloy

**DOI:** 10.1038/s41467-020-14641-1

**Published:** 2020-02-11

**Authors:** Sijing Chen, Hyun Seok Oh, Bernd Gludovatz, Sang Jun Kim, Eun Soo Park, Ze Zhang, Robert O. Ritchie, Qian Yu

**Affiliations:** 1 Department of Materials Science & Engineering, Center of Electron Microscopy and State Key Laboratory of Silicon Materials, Zhejiang University, 310027 Hangzhou, China; 2 Research Institute of Advanced Materials, Department of Materials Science and Engineering, Seoul National University, Seoul, 08826 Republic of Korea; 3 School of Mechanical and Manufacturing Engineering, UNSW Sydney, Sydney, NSW 2052 Australia; 4 Materials Sciences Division, Lawrence Berkeley National Laboratory, Berkeley, CA 94720 USA; 5 Department of Materials Science & Engineering, University of California, Berkeley, CA 94720 USA

**Keywords:** Mechanical properties, Metals and alloys

## Abstract

Strategies involving metastable phases have been the basis of the design of numerous alloys, yet research on metastable high-entropy alloys is still in its infancy. In dual-phase high-entropy alloys, the combination of local chemical environments and loading-induced crystal structure changes suggests a relationship between deformation mechanisms and chemical atomic distribution, which we examine in here in a Cantor-like Cr_20_Mn_6_Fe_34_Co_34_Ni_6_ alloy, comprising both face-centered cubic (*fcc*) and hexagonal closed packed (*hcp*) phases. We observe that partial dislocation activities result in stable three-dimensional stacking-fault networks. Additionally, the fraction of the stronger *hcp* phase progressively increases during plastic deformation by forming at the stacking-fault network boundaries in the *fcc* phase, serving as the major source of strain hardening. In this context, variations in local chemical composition promote a high density of Lomer-Cottrell locks, which facilitate the construction of the stacking-fault networks to provide nucleation sites for the *hcp* phase transformation.

## Abstract (web summary)

In dual-phase Cantor-like high entropy alloys, how local chemistry affects enhanced deformation mechanisms remains unclear. Here, the authors image 3D stacking fault networks formation and show they both impede dislocations and facilitate phase transformations via local chemical composition variations.

## Introduction

High-entropy alloys (HEAs), which were originally developed as concentrated solid solutions of multiple principal elements in equal, or near equal, atomic ratios^1^, have drawn progressively increasing attention over the past decade. Unlike single principal-element alloys, their complex alloy compositions and elemental distribution, together with crystal lattice distortion induced by atomic-size mismatch (depending upon relative atomic sizes)^2^, can have a significant effect on the activity of their crystal defects and hence mechanical properties^3–5^. Although HEAs were initially designed to benefit from single-phase stabilization^1,6–8^, single-phase HEAs are actually not that common, and of those that do exist, only a few display excellent damage-tolerance in the form of combinations of high strength, ductility, and toughness^8–10^. The well-studied equiatomic CrMnFeCoNi Cantor alloy^6^ and its derivatives, in particular the CrCoNi alloy^11^, show exceptional damage-tolerance which progressively improves at cryogenic temperatures, although even these alloys do not have yield (as opposed to ultimate tensile) strengths that are comparable to some advanced steels^6^. In light of this, certain strategies that involve the introduction of phase boundaries have been proposed to increase these mechanical properties of HEAs. For example, Li et al.^12^ reported a dual-phase high-entropy alloy (DP-HEA) that displays higher tensile yield strength and ductility than many single-phase HEAs (including the Cantor alloy). Subsequently, other DP-HEAs have been developed with excellent mechanical properties that exceed most traditional dual-phase alloys^13–16^. Different from many other dual-phase systems^17,18^, the *fcc* phase in these DP-HEAs can easily transform to the *hcp* phase through the glide of partial dislocations^16,19–21^; consequently, the volume fraction of the two phases can progressively change during plastic deformation^22^, resulting in a steady hardening effect with increasing strain under high stress. Therefore, studying the dynamic microstructural evolution and its effect on the deformation behavior of DP-HEAs is critical to the guidance and motivation of future research on the metastability and design of HEAs. Using molecular-dynamics simulations, Fang et al.^23^ emphasized the contribution of the *fcc*/*hcp* phase transformation, otherwise known as the transformation-induced plasticity (TRIP) effect, to the promotion of strength and plasticity in these HEAs. However, direct experimental evidence of the dynamic microstructure evolution particularly the phase transformation and its direct impact on mechanical behavior is still lacking, since most experimental studies to date have been relatively large-scale and have involved only post-mortem investigations of the structure and dislocation activity. With such DP-HEAs, as the local chemical environment varies everywhere, combined with a deformation-induced change in crystal structure, there is a scientific imperative to understand the relationships between deformation mechanisms in their dual-phase structure and the chemical distribution of atoms.

Here, we report on in situ transmission electron microscopy (TEM) observations on a DP-HEA, with a Cantor-like composition (in at.%) of Cr_20_Mn_6_Fe_34_Co_34_Ni_6_ that comprises an *fcc* austenite phase and an *hcp* ε-martensite phase, with the objective of discerning the fundamental origin of its enhanced strain hardening and the atomic-scale mechanisms by which this occurs. We find that the strain-hardening results from the formation of three-dimensional stacking-fault networks that impede dislocation motion and further provide preferred sites for the formation of the *hcp* phase via a TRIP effect, phenomena that appear to be promoted by variations in local chemical composition.

## Results

### Microstructure and tensile properties

**Fig. 1:**
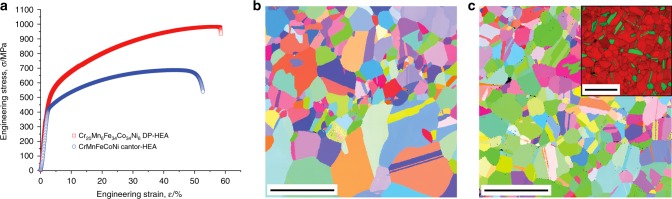
Comparison of the dual-phase and Cantor alloys. **a** Engineering uniaxial tensile stress–strain curves for the DP-HEA compared to that of the CrMnFeCoNi Cantor HEA. Corresponding EBSD maps (scale bar, 20 μm) of the microstructures of **b** the Cantor alloy and **c** the DP-HEA. The inset in **c** shows a phase map of the DP-HEA, where red represents the austenitic *fcc*-phase and green the *hcp* ε-martensite. The overall microstructure in the DP-HEA has a grain size that varies between ~3 and 10 μm.

The uniaxial tensile properties, in terms of engineering stress–strain curves, of the Cr_20_Mn_6_Fe_34_Co_34_Ni_6_ DP-HEA are compared to those of the equiatomic CrMnFeCoNi Cantor alloy in Fig. 1a. Details of the processing procedures, sample preparation, and testing methods are given below in the “Methods” section. Its microstructure, again compared with that of the Cantor alloy, is shown in Fig. 1b, c; the inset in Fig. 1c is a phase map of the DP-HEA, where red represents the austenitic *fcc*-phase and green the *hcp* ε-martensite.

### In situ TEM-straining tests

As shown in Fig. 1a, the DP-HEA displays a remarkable combination of strength and ductility with a tensile strength approaching 1 GPa and a tensile ductility of almost 60% at ambient temperatures, properties which are substantially higher than many single-phase HEAs, including the Cantor alloy. In order to discern the role of the constituent two phases and the salient deformation mechanisms, the DP-HEA was investigated using several nanoscale techniques, including in situ straining tests and quantitative in situ compression tests in the TEM, state-of-the-art spherical aberration-corrected scanning transmission electron microscopy (STEM) and energy-dispersive x-ray spectroscopy (EDS) with atomic resolution. Although deformation-induced phase transformations are known to occur in DP-HEAs^12–16^, our current in situ TEM studies demonstrate that the phase transformation from *fcc* to *hcp* is based on the formation of three-dimensional (3D) stacking-fault networks comprising multiple stacking faults (SFs) and sessile Lomer–Cottrell locks, which we believe are promoted by intrinsic chemical variation in HEAs. We further employ nanoscale compression pillar testing to establish the mechanisms underlying how this continuous *fcc* → *hcp* phase transformation plays a dominant role in generating the significant strain hardening in this HEA.

**Fig. 2:**
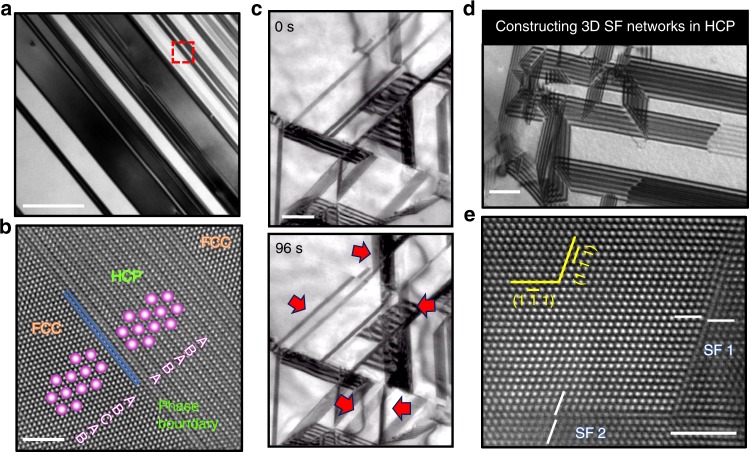
TEM and STEM characterization of the DP-HEA. **a** Bright-field (BF) TEM image of the DP morphology (scale bar, 200 nm). **b** High-resolution HAADF-STEM image of a small area in the red box in Fig. 2a, showing a typical *hcp* thin plate in the *fcc* matrix (scale bar, 2 nm). **c** TEM images represent the dynamic process of the generation of 3D stacking-fault networks in the *fcc* matrix (shown in real time in Supplementary Movie 2) (scale bar, 500 nm). The red arrows point to several immobile stacking faults newly formed during the in situ deformation. **d** Similar 3D stacking-fault networks formed in the *hcp* structure (scale bar, 200 nm). **e** HAADF image of a Lomer–Cottrell lock that formed by the reaction of two partial dislocations, with stacking faults SF1 and SF2, on two different {111} slip planes (scale bar, 2 nm).

Microstructure characterization and composition analyses were carried out by TEM and high-angle annular dark field-STEM (HAADF-STEM), as shown in Fig. 2. The bright-field TEM image in Fig. 2a demonstrates that the alloy is composed of an *fcc* matrix in which the *hcp* phase grows in the form of laminate with a thickness ranging from several to several hundred nanometers. High-resolution HAADF-STEM images of the *hcp* phase were taken along the *fcc* [110] direction (Fig. 2b), from which the coherent phase boundaries between the *fcc* and *hcp* phases can be observed. The interphase plane is represented by the close-packed planes of each structure: (111) for the *fcc* and (0001) for the *hcp* phase. No crystal defects, such as dislocations could be observed at these phase boundaries.

Compared to other single-phase HEAs, the microstructure of this DP-HEA is significantly more complex. The *hcp* phase grows in the *fcc* matrix and, as such, serves to terminate slip planes for dislocation glide, thereby reducing the mean-free path for dislocation glide. In addition, the activation energy for dislocation slip in the *hcp* ε-martensite should be higher than that in the *fcc* phase^24^. To examine these effects directly, we used a set-up in the TEM that allows real-time observations of the dynamic evolution of the activity of defects. Specifically, the active plasticity mechanisms were investigated during deformation at ambient temperatures using a Gatan 654 single-tilt straining holder in a FEI Tecnai G2 F20 TEM operating at 200 kV. For example, Supplementary Movie 1 was recorded during the early stage of deformation and shows the easy motion of partial dislocations in the *fcc* phase. This is similar to behavior that we reported previously for the CrMnFeCoNi Cantor alloy^25^. According to our observations over a wide range of strains, the fast movement of the partial dislocations dominates the deformation process at the start of plastic deformation and provides for initial deformability (ductility). In some cases, the trailing partial catches up with the leading partial to remove the intervening SF and relieve the local stresses. Images captured from Supplementary Movie 2 in Fig. 2c further illustrate that in other cases, partial dislocation activity, which dynamically controls the initial plastic deformation, can result in frequent interactions with SFs to create sessile dislocation junctions that act to significantly inhibit local dislocation motion and hence cause strain hardening^26,27^. In turn, the planar SFs between the dissociated dislocation defects form into 3D volume defects through connections via Lomer–Cottrell locks (discussed below) to develop into SF networks that are widely observed throughout the *fcc* phase. In Fig. 2c, the red arrows indicate several newly formed immobile SFs that became part of the 3D stacking-fault network. Similar SF networks form in the *hcp* phase as well, as shown in Fig. 2d. The HAADF-STEM image in Fig. 2e shows the real space atomic structure of such a partial dislocation reaction in the current DP-HEA. The image was taken along the [110] zone axis and shows two dislocations with Burgers vectors of *a*/2[101] and *a*/2[0$\bar 1\bar 1$] on (1$\bar 1\bar 1$) and (1$\bar 1$1) planes, respectively. The dislocation reactions were determined to be

1 $$\frac{a}{2}\left[ {101} \right] \to \frac{a}{6}\left[ {211} \right] + \frac{a}{6}[1\bar 12],$$

2 $$\frac{a}{2}[0\bar 1\bar 1] \to \frac{a}{6}[1\bar 1\bar 2] + \frac{a}{6}[\bar 1\bar 2\bar 1].$$

The interaction of partial dislocations then results in the creation of an *a*/6[1$\bar 1$0] stair-rod dislocation through:

3 $$\frac{a}{6}[211] + \frac{a}{6}[\bar 1\bar 2\bar 1] \to \frac{a}{6}[1\bar 10].$$

The resultant *a*/6[1$\bar 1$0] dislocation is immobile because it is not formed on an active slip plane. Such sessile dislocations, that are generated in the *fcc* matrix of the DP-HEA, are known as Lomer–Cottrell locks^28^. They act to immobilize dislocation motion on the slip planes, thereby leading to an enhanced work-hardening rate. Indeed, the Lomer–Cottrell locks formed during deformation provide several important benefits to the mechanical properties of the dual-phase alloy: they serve to stabilize the stacking-fault network, which consequently presents a strong hindering effect on dislocation motion (work hardening) and additionally promote the nucleation and growth of the *hcp* phase at the base of the SFs.

**Fig. 3:**
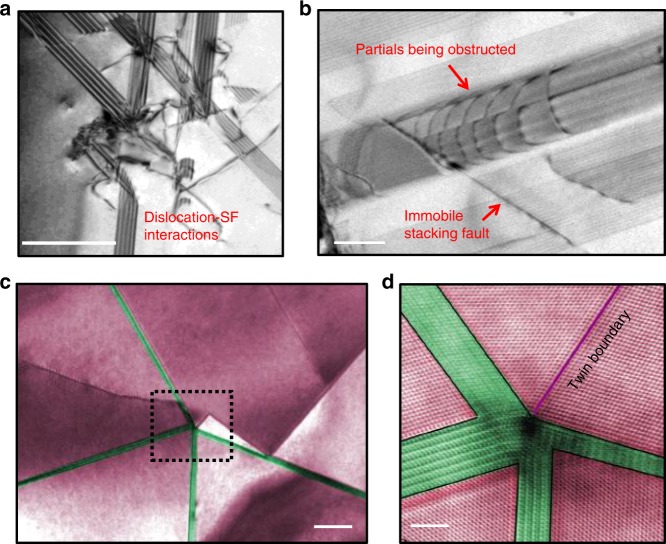
Intersection of thin *hcp* plates and obstacle effect of stacking faults on dislocation motion. **a** Bright-field TEM image showing the blocking of dislocations by the 3D stacking-fault network (scale bar, 500 nm). **b** Partial dislocations being hindered by an immobile stacking fault, which eventually reversed the dislocation glide direction (scale bar, 200 nm). **c** STEM image of a conjunction of four *hcp* lamellae and one twin (scale bar, 20 nm). **d** Higher magnification HAADF image corresponding to the region marked by the box in **c** (scale bar, 2 nm).

Specifically, the Lomer–Cottrell locks produce a high density of immobile SFs upon which the stable SF networks are constructed. Each immobile SF can act as strong barrier to hinder dislocation motion. As shown in Fig. 3a, b, dislocations tend to pile-up in front of the immobile SFs. Although it is difficult to quantitatively calculate the interaction energy, it is clear that the immobile SFs are difficult for dislocations to penetrate.

Additionally, the Lomer–Cottrell locks also link and stabilize the SFs; this in turn promotes the nucleation and growth of the *hcp* phase at the base of the stabilized SFs. They appear to guide dislocation motion parallel to the immobile SFs in the SF networks and thereby encourage the phase transition from the *fcc* to *hcp* phase. SFs that are so stabilized by Lomer–Cottrell locks provide reliable platforms for the *hcp* phase to form and grow. When leading Shockley partial dislocations, with Burgers vector 1/6〈112〉 gliding on {111} planes, become obstructed at a Lomer–Cottrell lock with dislocations of the same Burgers vector gliding on every other plane, the local structure changes from ABCABC stacking sequence of the *fcc* structure to the ABAB sequence of the *hcp* structure. This in situ phase transformation under load (TRIP effect) is shown in real time in Supplementary Movie 3, where thin plates of the *hcp* structure are formed by successive glide of partial dislocations on {111} planes. The *hcp* ε-martensite preferentially nucleates at the intersection of the SFs in the networks in the form of thin plates with thickness of only few nanometers^29^. In essence, the SF networks supply embryos for the phase transformation to occur. As illustrated in Fig. 3c, the intersection of four nano-sized *hcp* lamellas and one twin are shown at the quadruple junction of SFs. The corresponding higher magnification HAADF image of this feature in Fig. 3d further shows that the strain field at the junction is very narrow and localized at the center; this indicates that little lattice mismatch and strain energy was introduced due to the interaction of phases with each other and with defects such as the twin.

### Nanoscale pillar compression tests

**Fig. 4:**
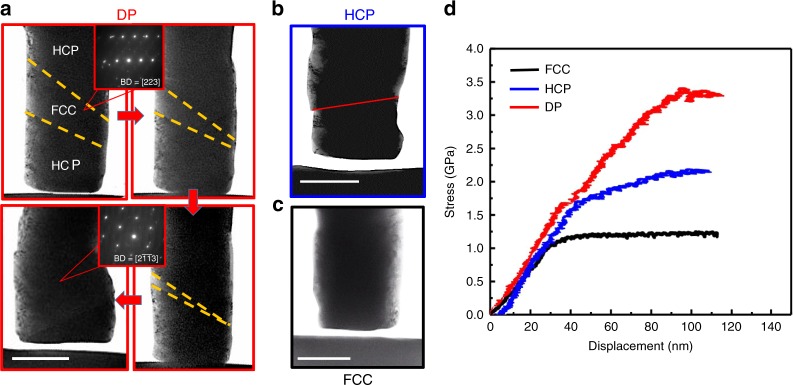
In situ TEM compression tests on three pillars with the *fcc* austenite, the *hcp* -martensite and the *fcc/hcp* dual-phase structures, respectively. **a** High-resolution TEM images captured from Supplementary Movie 4 (scale bar, 200 nm) of the DP pillar, wherein the *hcp* phase continues to grow at the expense of the *fcc* phase. **b** TEM image of a representative *hcp* pillar after compression; the red dashed line indicates the location of localized slip (scale bar, 200 nm). **c** TEM image of a representative *fcc* pillar after compression (scale bar, 200 nm). **d** Engineering stress-displacement curves of the three pillars in the in situ TEM compression test.

The formation of an *hcp* phase from the SF network is important as with further deformation, the thin *hcp* plates tend to grow on these networks which plays a critical role in accommodating deformation. To quantitatively analyze the contribution to the mechanical properties from this increasing volume fraction of the *hcp* phase, ~300-nm diameter pillars of the *fcc* austenite, the *hcp* ε-martensite and the *fcc/hcp* dual-phase structures were prepared and compressed (further details are given in the “Methods” section). A relatively large grain was chosen so that the *fcc, hcp*, and *fcc*/*hcp* pillars were made from the same grain to maintain the same orientation. Three representative engineering stress-displacement curves from these compression tests are plotted in Fig. 4d, where the stress was calculated by dividing load with the real-time contact area. (Note that threshold load values for machine noise were measured to be around −1 to 1 μN). Distinct from traditional metals tested with similar dimensions, all three pillars deformed relatively homogeneously, indicating high plastic stability. The higher lattice friction in HEAs associated with their multiple principal-element compositions, as compared to traditional dilute solid-solution alloys, is reasoned to be important for enhancing dislocation pinning and reducing the surface escape of dislocations. For the three structures, the dual-phase pillar displayed the highest ultimate strength at ~3.45 GPa, followed by the pure *hcp* pillar which showed an ultimate strength of ~2.25 GPa; the corresponding pure *fcc* pillar has the lowest ultimate strength of only ~1.25 GPa, effectively only one-third of that of the DP pillar. Clearly, the *hcp* phase is distinctly stronger than the *fcc* phase, consistent with the macroscale stress–strain curves in Fig. 1a. Moreover, as the yield strength of the DP and *hcp* pillars are quite similar, the work-hardening rate of the dual-phase structure is clearly much higher than that shown by the individual *hcp* and *fcc* phases. We believe that the higher work-hardening rate in the DP pillars during these compression tests can be directly associated with the in situ transformation from the growth of the *hcp* phase within the *fcc* matrix. The behavior of one DP-HEA pillar during deformation is given in Supplementary Movie 4, and provides a real time demonstration of this TRIP effect in the dual-phase alloy. Snapshots shown in Fig. 4a indicate that the phase boundaries in this DP pillar are stable during the early stage of the compression test, but at the yield point, one such boundary starts to migrate such that the *hcp* domain continues to grow at the expense of the *fcc* domain in the middle of the pillar. Such a continuous increase in the *hcp*/*fcc* ratio with deformation sustains the hardening effect with the flow stress increasing two-fold from 1.75 to ~3.5 GPa; this is a far higher rate (in terms of the slope of the mechanical curve after yielding) than either of the single-phase pillars, and represents an outstanding hardening effect. Throughout the entire deformation process, the *hcp* phase deforms essentially homogeneously until plastic instability ensues via catastrophic localized shear at ~3.5 GPa. Although we were unable to observe the detailed dislocation activity during the compression test, we would presume that the growth of *hcp* phase, manifest through the migration of the phase boundary, was achieved by the glide of partial dislocations^12,30^. We tested more pillars in which the phase boundary was almost perpendicular to the loading direction (Supplementary Movie 5). In this case, the boundary can still hinder dislocation motion but there was no increase in the volume fraction of *hcp*; we observed the stress-displacement curve to remain essentially flat after yielding (see Supplementary Fig. 1), indicating that the direct contribution of the hindering effect of boundaries to hardening is quite limited, as compared to the continuous phase transformation. Therefore, we concluded that the more important role of the stacking-fault networks may be to supply embryos for the *fcc* → *hcp* phase transformation.

We believe that the stable stacking-fault networks are the foundation of the dynamic phase transformation (TRIP) effect, which is the primary origin of plasticity and exceptional strain hardening of this dual-phase (Cantor-like) high-entropy alloy. In contrast, although SFs are prevalent in traditional metals and alloys with low stacking-fault energies, the creation of stable 3D stacking-fault network structures at the early stage of plastic deformation in the DP-HEAs appears to be quite unique. The formation of these networks can be directly related to the presence of a high density of immobile Lomer–Cottrell locks. The intentional generation of such locks to promote work-hardening raises a fundamental question of how to increase the probability of interactions between dissociated dislocations with the sole objective of enhancing the formation of such locks.

### Variations in local chemical environment

**Fig. 5:**
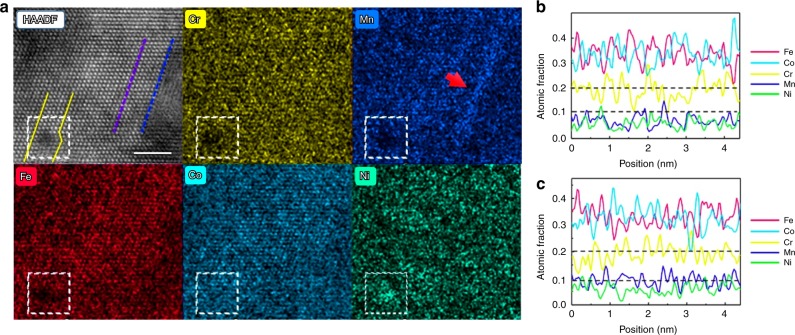
Aberration-corrected STEM imaging, mapping, and line profiles of elemental distributions at the intersection of two stacking faults in the DP-HEA. **a** HAADF image of the junction of two SFs taken along the [110] zone axis and EDS maps for individual elements of Cr, Mn, Fe, Co, and Ni, displaying the segregation of Ni and Mn at the SFs (scale bar, 2 nm). **b** Line profiles represent the atomic fraction of individual elements taken from corresponding EDS maps in **a**, which show the variation in elements for columns of atoms on the ($1\bar 1\bar 1$) plane projected along [110] beam direction along the purple line. **c** Line profiles along the blue line, which is located at the SF.

In our in situ TEM studies, although the motion of the partial dislocations was certainly hindered by the presence of the phase boundaries and SF structures, at the onset of plastic deformation it was often observed that moving partial dislocations would stop in the middle of the matrix as if an invisible obstacle were present. In view of the mixture of elements in HEAs, one such “invisible” obstacle could be related to the existence of local variations in chemical composition, as such variations would modify the local lattice environment (elastically and electronically) and hence the nature of dislocation behavior^31,32^, of which a typical example is the multiplication of dislocations. To investigate this notion, we examined the local chemical environment around the SFs using large solid-angle EDS focused on the SF junction in an aberration-corrected Titan G2 STEM FEI (operating at 200 kV). Figure 5a shows the resulting HAADF image and atomic-resolution EDS maps along the [110] zone axis. Line profiles in Fig. 5b, c represent the content variation of elements for columns on the (1$\bar 1\bar 1$) plane projected along [110] beam direction. Ni is the most unevenly distributed element of the five; its atomic fraction fluctuates from −58% to 115% about its average. Mn shares a similar degree of inhomogeneity with Ni, whereas Fe, Co, and Cr display far less chemical undulation. The line profiles in Fig. 5b, c show the density variation of all five elements along the purple and blue dashed lines in Fig. 5a, respectively; the blue line was located in the vicinity of the SF and the purple line was in the matrix of the *fcc* phase. These line profiles demonstrate that the average atom fraction of Mn near the SF is nearly 30% higher than that in the matrix, while the other four elements did not display such differences. The EDS maps that we obtained have very high signal-to-noise ratio, due to the high-quality atomic resolution EDS mapping (details are presented in the “Methods” section); additionally, the fluctuation of the concentration depends on the difference in concentration between one atomic column and its adjacent atomic columns, not the absolute value of the concentration. We also compared the composition fluctuation in a Ni–3W alloy and a Ni-based complex alloy to show the variation in local concentrations with elements. As shown in Supplementary Fig. 2, in Ni–3W the variation in the Ni concentration is relatively small, ~3%, while the local concentration of Ni varies from ~70% to ~50% in the Ni-based complex alloy. These results are consistent with the recent experimental observations and simulations that suggest the distribution of the elements in HEAs is not homogeneous at the lattice scale; concentration waves exist intrinsically and universally, which may make these multiple principal-element alloys distinct from more traditional alloys^33–35^. In this work, over 15 sites were investigated; it is suggested that at the core of the partial dislocations where the SFs end, the concentration of one element significantly increases, while the exact local compositions may vary case by case. For instance, in Fig. 5a, the local chemical environment in this sub-nanometer region was found to be enriched in Ni. It is worth noting that although this dual-phase HEA contains the same five elements as the Cantor alloy, the fluctuation in local concentration of elements is much higher. It is also reasoned that there is larger lattice distortion in the dual-phase HEA Cr_20_Mn_6_Fe_34_Co_34_Ni_6_ than in the Cantor alloy, with the increase in Co and Fe content and reduction in Mn and Ni content^31^. Such variations in chemical composition not only create physical obstacles to dislocation glide at the atomic scale but also may be a key factor in the adjustment in the local stacking-fault energy. For example, if we consider a locally enhanced concentration of Ni (Fig. 5a) and Mn (Fig. 5c), according to our thermodynamic calculations using Thermo-calc software along with the TCFE8 database, the differences between chemical Gibbs free energies of the *hcp* and *fcc* phases at 300 K are −73.9 J/mol for the matrix composition, 47.3 J/mol for the Mn-segregated composition (30% higher Mn composition (Cr_20_Fe_34_Co_34_Ni_6_)_97.9_Mn_8_), and −27.5 J/mol for the Ni-enriched composition (Cr_18_Mn_4_Fe_30_Co_34_Ni_14_)). In these cases, the segregated compositions would have relatively higher SFE as the Gibbs free energy difference is the chemical origin of the stacking-fault energy^21^.

## Discussion

In summary, our results demonstrate that in the dual-phase Cantor-like high-entropy alloy, it is the continuous in situ phase transformation that provides the dominant contribution to strain hardening. As distinct from traditional dual-phase alloys, in the DP-HEAs the motivation for this transformation lies in the creation of stacking-fault networks, which seed the *fcc* → *hcp* transformation and which are intrinsically formed because of the fluctuation in lattice friction associated with concentration waves. However, it is worth noting that the fracture toughness of these TRIP HEAs may not be similarly enhanced compared to the Cantor alloy, particularly at decreasing temperatures. Despite the stress-induced TRIP effect of the *fcc* → *hcp* transformation, which acts to enhance the degree of strain hardening to the benefit of strength and ductility, fracture toughness is also a function of the inherent toughness of the product *hcp* phase, and this ε-martensite phase, despite its strength, tends to be quite brittle and not characterized by any significant degree of toughness.

However, although there is still much to explain in the mechanical behavior of dual-phase HEAs, we believe that our study can provide a sound foundation for the understanding of their local chemical structure at the atomic scale and the synergetic effect with the dynamic evolution of microstructure from the TRIP effect, which underlies the resulting structure–properties relationships in this complex Cantor-like dual-phase alloy.

## Methods

### Alloys processing

Using pure metals with a purity better than 99.8%, 4-kg ingots swere cast in a vacuum induction furnace. The as-cast ingots were hot rolled at 900 °C, with a rolling reduction ratio of 50%. The hot rolled samples were homogenized for 3 h at 1200 °C followed by water quenching. The homogenized samples were then cold rolled to 40–50% thickness reduction before undergoing recrystallization annealing under an air atmosphere at 800 °C for 20 min for the Cantor alloy and 800 °C for 1 h for the DP-HEA, followed by water-quenching.

### Mechanical testing

Flat specimens, with a thickness of 1 mm, were employed for uniaxial tensile testing; these were sectioned from the recrystallized samples near the surface along the TD direction using electrical discharge machining (EDM). An oxidation layer that occurred during EDM cutting was subsequently removed carefully using mechanical grinding. The gauge length and width of the tensile specimens were 10.2 and 3.2 mm, respectively. Uniaxial tensile tests were carried out at room temperature on an Instron 5967 universal testing machine (Instron, Norwood, USA) at the strain rate of 1 × 10^−3^ s^−1^. The strain evolution during the tensile tests was monitored by the displacement transducer on the testing machine, but was accurately measured during testing using an Advanced Video Extensometer camera and digital image correlation techniques. At least three samples for each material were tested to confirm reproducibility.

### Characterization methods

Microstructure characterization and composition analyses were carried out by TEM, HAADF-STEM, and large solid angle EDS. The EDS mapping was performed using an aberration-corrected scanning transmission electron microscope (STEM, FEI Titan Cubed Themis G2 300) operated at 300 kV with a convergence semi-angle of 23.6 mrad and equipped with a DCOR plus spherical aberration corrector for the electron probe which was aligned prior every experiment using a gold standard sample. The beam current was set between 25 and 30 pA. The dwell time was 1 µs per pixel with a map size of 256 × 256 pixels; a complete process of EDS mapping took roughly 1.5 h to reach a high signal-to-noise ratio. The benchmark systems of Al_2_O_3_ and NiTi were tested to make sure that the EDS system that we used had a sufficiently high accuracy of composition measurement. The measurement results of Al_2_O_3_ demonstrated a ratio of the atomic percentage of Al:O = 39.78:60.22; for NiTi, the ratio is Ni:Ti = 50.16:49.84. The software that we used to quantify and analyze the EDX data was Velox, which is available from the FEI Company. Specifically, the behavior of defects during plastic deformation was studied by in situ TEM-straining tests. By using a Gatan 654 single-tilt straining holder, the uniaxial straining tests were performed at ambient temperature in a FEI Tecnai G2 F20 TEM (operating at 200 kV). The tensile loading rate was ~1 μm/s. Time-resolved TEM and high-resolution TEM images of the regions of interest were recorded with a Gatan CCD camera at 10 frames per second. The regions closest to the hole were the thinnest, which were monitored during the straining process. In situ compression experiments were carried out in an FEI Tecnai G2 F20 TEM (operating at 200 kV) with Hysitron Pi95 TEM nanoindentor in displacement-control mode and the displacement rate was 2 nm/s. Micropillars for the compression tests were prepared using FEI Quanta 3D FEG focus ion beam (FIB) micro-machining technique by Ga+ ion beam and the diameter of the cross-section was about 300 nm. We performed calibration before nano-compression; the threshold load values for machine noise was ~ −1 to 1 μN.

### Sample preparation

Specimens for in situ straining tests were first sectioned from the alloy plates using electric discharge machining followed by polishing with SiC papers down to thickness of 80 μm. In order to achieve electron-transparent observation regions, the samples were further thinned by using jet polishing until a hole appeared in the middle of the foils, after which the specimens were attached to stainless-steel substrates with narrow rectangular windows for transmission of the electron beam. Only samples that were well attached to the substrate, free of contamination from the sample preparation procedure which did not rotate or bend, were selected for detailed TEM investigation.

## Supplementary information

Supplementary Information

Description of Additional Supplementary Information

Supplementary Movie 1

Supplementary Movie 2

Supplementary Movie 3

Supplementary Movie 4

Supplementary Movie 5

## Data Availability

All data generated or analyzed during this study are included in the published article and are available from the corresponding authors upon reasonable request.
