# Supplementary material for: Real-time observations of TRIP-induced ultrahigh strain hardening in a dual-phase CrMnFeCoNi high-entropy alloy
Source: Nat Commun. 2020 Feb 11;11:826. doi: 10.1038/s41467-020-14641-1 (PMC7012927; doi:10.1038/s41467-020-14641-1)
Supplement: Supplementary file 1 — Supplementary Information [file 41467_2020_14641_MOESM1_ESM.pdf]

Supplementary Information

Real-time observations of TRIP-induced ultrahigh strain hardening in a dual-phase CrMnFeCoNi high-entropy alloy

Sijing Chen[1], Hyun Seok Oh[2], Bernd Gludovatz[3], Sang Jun Kim[2], Eun Soo Park[3], Ze Zhang[1], Robert O. Ritchie[4,5*] and Qian Yu[1*]

[1]Department of Materials Science & Engineering, Center of Electron Microscopy and State Key Laboratory of Silicon Materials, Zhejiang University, Hangzhou 310027, China

[2]Research Institute of Advanced Materials, Department of Materials Science and Engineering, Seoul National University, Seoul 08826, Republic of Korea

[3]School of Mechanical and Manufacturing Engineering, UNSW Sydney, NSW 2052, Australia

[4]Materials Sciences Division, Lawrence Berkeley National Laboratory, Berkeley, CA 94720, USA

[5]Department of Materials Science & Engineering, University of California, Berkeley, CA 94720, USA

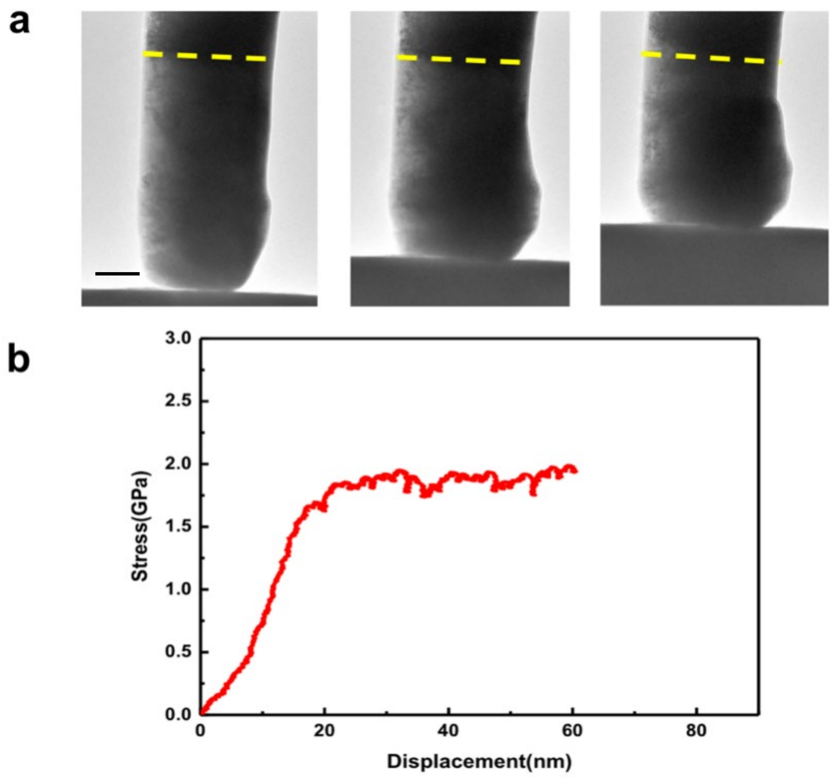

Supplementary Fig. 1. ***In situ*** **TEM compression tests on a pillar with the** ***fcc/hcp*** **dual-phase structure, where the** ***fcc/hcp*** **phase boundary was almost perpendicular to the loading direction.** There was no increase in the volume of the *hcp* phase. **a** TEM images captured from Supplementary Movie 5 of the DP HEA pillar (scale bar, 100 nm). **b** Engineering stress-displacement curve of the DP HEA pillar.

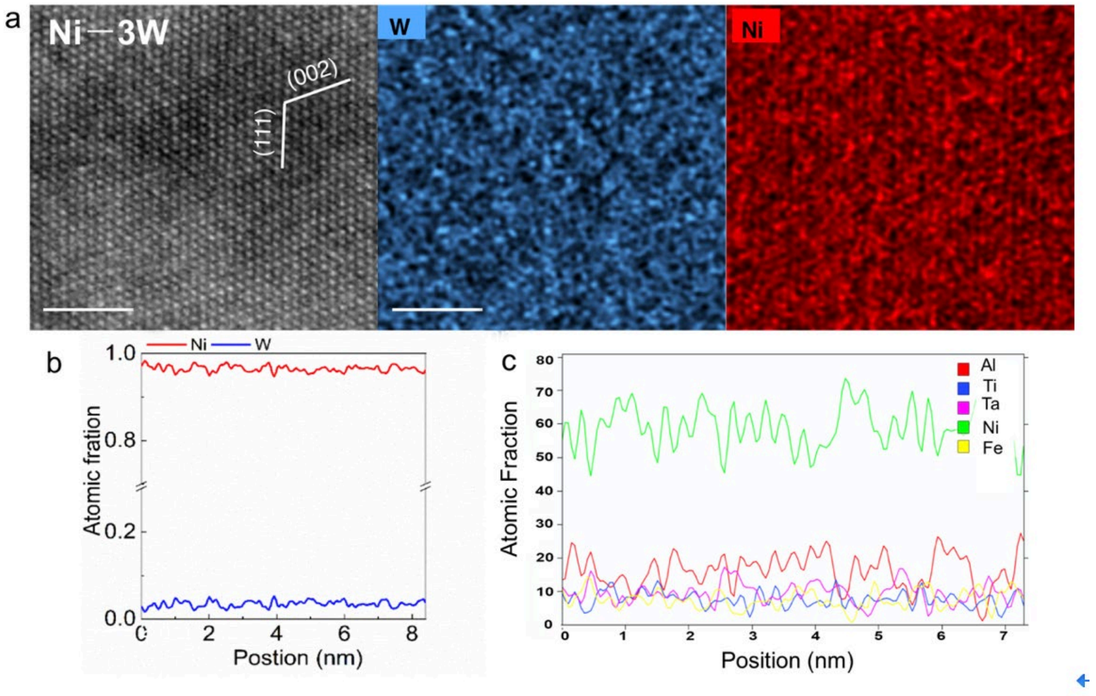

Supplementary Fig. 2. EDS mapping and concentration fluctuations analysis in a Ni-3W alloy and a Ni-based complex superalloy. **a** EDS maps of Ni and W in a Ni-3W alloy. Scale bars represent 2 nm. **b** Typical line profile of the concentration fluctuations along [002] in the Ni-3W alloy. **c** Typical line profile of the concentration fluctuations along [002] in a Ni-based complex superalloy. The fluctuation of element concentrations is far higher compared to that in Ni-3W.
