# Supplementary material for: Real-time observations of TRIP-induced ultrahigh strain hardening in a dual-phase CrMnFeCoNi high-entropy alloy
Source: Nat Commun. 2020 Feb 11;11:826. doi: 10.1038/s41467-020-14641-1 (PMC7012927; doi:10.1038/s41467-020-14641-1)
Supplement: Supplementary file 2 — Description of Additional Supplementary Information [file 41467_2020_14641_MOESM2_ESM.pdf]

**Description of Additional Supplementary Files**

File Name: Supplementary Movie 1: Easy motion of partial dislocations.
Description: At the early stage of deformation, the nucleation and movement of partial dislocations in the *fcc* phase dominate the plastic deformation.

File Name: Supplementary Movie 2: Dynamic stacking-fault network
Description: Formation of a dynamic three-dimensional stacking-fault network by frequent interaction of dislocations with stacking faults in the *fcc* matrix. The stacking-fault network exists in a state of constant change, making it a flexible regulation on dislocation movement.

File Name: Supplementary Movie 3: Formation of the *hcp* phase from the glide of partials.
Description: Partial dislocations, nucleated at the intersection of the stacking faults, which glide successively on {111} planes in the *fcc* phase, forming an embryo of the *hcp* phase.

File Name: Supplementary Movie 4: *In situ* compression test on a DP-HEA pillar.
Description: *In situ* TEM nano-compression on a dual-phase high-entropy alloy pillar that comprises of both the *hcp* phase and the *fcc* phase. The *hcp* domain continues to grow at the expense of the *fcc* domain in the middle of the pillar during compression.

File Name: Supplementary Movie 5: *In situ* compression test on a DP-HEA pillar in which the phase boundary was perpendicular to the loading direction.
